# Impact of body mass index on in-hospital mortality in older patients hospitalized for bacterial pneumonia with non-dialysis-dependent chronic kidney disease

**DOI:** 10.1186/s12877-022-03659-3

**Published:** 2022-12-09

**Authors:** Akira Okada, Satoko Yamaguchi, Taisuke Jo, Isao Yokota, Sachiko Ono, Kayo Ikeda Kurakawa, Masaomi Nangaku, Toshimasa Yamauchi, Takashi Kadowaki

**Affiliations:** 1 Department of Prevention of Diabetes and Lifestyle-Related Diseases, Graduate School of Medicine, The University of Tokyo, Tokyo, Japan; 2 Department of Respiratory Medicine, Graduate School of Medicine, The University of Tokyo, Tokyo, Japan; 3 Department of Health Services Research, Graduate School of Medicine, The University of Tokyo, Tokyo, Japan; 4 Department of Biostatistics, Graduate School of Medicine, Hokkaido University, Sapporo, Hokkaido Japan; 5 Department of Eat-Loss Medicine, Graduate School of Medicine, The University of Tokyo, Tokyo, Japan; 6 Division of Nephrology and Endocrinology, Graduate School of Medicine, The University of Tokyo, Tokyo, Japan; 7 Department of Diabetes and Metabolism, Graduate School of Medicine, The University of Tokyo, Tokyo, Japan; 8 Toranomon Hospital, Tokyo, Japan

**Keywords:** Clinical epidemiology, Underweight, Kidney function, Body mass index, Pneumonia

## Abstract

**Background:**

Low body mass index (BMI) in older individuals with decreased kidney function is important because of its association with poor prognosis and frailty. Herein, we aimed to clarify the association between BMI and in-hospital mortality among older patients with non-dialysis-dependent chronic kidney disease (CKD) stratified by kidney function.

**Methods:**

Using data from the Medical Vision Database, this multicentre cohort study included people aged ≥ 60 years with an estimated glomerular filtration rate of < 60 ml/min/1.73 m^2^ but without dialysis dependency, hospitalised for bacterial pneumonia during 2014–2019. We compared the risk of in-hospital death between patients with BMI categories based on the quartiles (low, medium–low, medium–high, and high) setting medium–high BMI as a reference. We further assessed the association with BMI using a cubic spline, setting BMI as a nonlinear continuous variable and a BMI of 22 kg/m^2^ as a reference. We also evaluated the association between BMI and kidney function using a generalised additive model adjusted for interaction terms between nonlinear continuous BMI and kidney function.

**Results:**

We obtained data for 3,952 patients, with 350 (8.9%) in-hospital deaths. When compared with medium–high BMI, low BMI was associated with an increased risk of death and longer hospital stay, whereas the other two categories were comparable. Models using a cubic spline showing an association between BMI and in-hospital death showed an L-shaped curve; BMI < 22.0 kg/m^2^ was associated with an increased risk for mortality, and at a BMI of 18.5 kg/m^2^, the odds ratio was 1.43 with a 95% confidence interval of 1.26–1.61 when compared with a BMI of 22.0 kg/m^2^. Analysis of the interactive effects of kidney function using the generalised additive model showed that a protective association of high BMI tapered along with decreased kidney function.

**Conclusions:**

This cohort study suggests not only that lower BMI and low kidney function are associated with in-hospital mortality independently but also that the protective effects of high BMI weaken as kidney function decreases via the analysis of the interaction terms. This study highlights the necessity for the prevention of underweight and demonstrates the interaction between BMI and kidney function in older patients with non-dialysis-dependent CKD.

**Supplementary Information:**

The online version contains supplementary material available at 10.1186/s12877-022-03659-3.

## Background

Obesity or overweight, defined by high body mass index (BMI), is associated with several diseases, including cardiovascular and metabolic diseases. High BMI is also closely associated with chronic kidney disease (CKD). Accumulated evidence from epidemiological studies has shown that obesity is associated with the development of proteinuria [1], kidney dysfunction [2], and conditions necessitating kidney replacement therapy [3]. At the same time, low BMI is also problematic in patients with CKD because low BMI is associated with an accelerated decrease in kidney function [4]. Furthermore, the relationship between low BMI and prognosis in older people has been emphasised [5], while decreased kidney function, which is more prevalent as age advances [6], is associated with frailty in older individuals [7]. Thus, both low BMI and low kidney function in older patients are important issues.

Albeit there is solid evidence that a higher BMI is associated with a better prognosis in patients on regular dialysis [8, 9], the association between BMI and prognosis among non-dialysis-dependent CKD patients is controversial. For example, a population-based study showed a U-shaped relationship between BMI and hazard ratios for mortality [10], and another cohort study showed that patients with a lower BMI had higher mortality owing to infection complicated by non-dialysis-dependent CKD [11]. Conversely, BMI was reportedly not associated with mortality in a similar population [12]. A meta-analysis suggested that the obesity paradox might be present in patients with CKD without dialysis dependency, despite a high level of heterogeneity [13]. Furthermore, most cohort studies on patients with CKD did not focus on the prognosis of patients hospitalised for acute diseases [10–12, 14, 15], while only one study suggested a U-shaped relationship between BMI and prognosis in patients hospitalised for acute myocardial infarction [16]. Thus, clinical epidemiological evidence of the association between BMI and in-hospital mortality among such patients is insufficient. Therefore, it remains unclear whether a higher BMI is beneficial for people, especially older individuals with CKD without dialysis dependency who are admitted for other acute diseases.

In this study, we aimed to clarify the effect of kidney function on the association between BMI and in-hospital mortality in older patients with non-dialysis-dependent CKD admitted for bacterial pneumonia, a communicable and acute-onset disease. A meta-analysis confirmed that a high BMI was associated with a good prognosis in patients admitted for pneumonia; however, this meta-analysis failed to adjust for kidney function or stratify the population by CKD stages [17]. In this study, we also aimed to stratify the population according to CKD stages in association with in-hospital mortality and BMI. Furthermore, we aimed to describe the interaction between kidney function and BMI for in-hospital mortality and length of hospital stay.

## Patients and methods

### Data source

We used data from a commercially available hospital-based database from Medical Data Vision (MDV), Tokyo, Japan. The MDV database contains claims data provided by hospitals nationwide using the Japanese Diagnosis and Procedure Combination (DPC) reimbursement system [18]. Currently, the DPC system is adopted in approximately 90% of all acute-care hospitals in Japan, and medical practices in the DPC hospitals reflect acute-care medicine in Japan [19].

The database contains data on characteristics of hospitalised patients: age, sex, body height, body weight, smoking status, and prognostic factors for pneumonia (information regarding the presence of dehydration, respiratory failure, orientation disturbance, immunosuppression, pulmonary consolidation, hypotension, and whether pneumonia was community-acquired pneumonia or nursing-/healthcare-associated pneumonia) as defined by the Japanese Respiratory Society [20]. Respiratory failure was classified into three categories: no respiratory failure, defined as percutaneous oxygen saturation (SpO_2_) > 90% on room air without oxygen administration; moderate respiratory failure, SpO_2_ ≤ 90% necessitating oxygen administration on a fraction of inspired oxygen < 35% to maintain SpO_2_ > 90%; and severe respiratory failure, SpO_2_ ≤ 90% necessitating oxygen administration on a fraction of inspired oxygen ≥ 35% to maintain SpO_2_ > 90%. An immunosuppressive state was defined as the presence of malignancy as a comorbidity or the use of immunosuppressive agents. Hypotension on admission was defined as systolic blood pressure ≤ 90 mmHg. The presence of pulmonary consolidation was recorded, defined as a binary value whose positivity means that pneumonia lesions accounted for ≥ two-thirds of one lung or a C-reactive protein level of ≥ 20 mg/dL [21]. The database includes information on in-hospital procedures performed, drugs administered, and disease diagnoses recorded using the International Classification of Diseases, 10th revision (ICD-10) codes [19].

This study was approved by the local research ethics board. Because of the anonymous nature of the data, the requirement for informed consent was waived.

### Study design and population

Using the MDV database, we extracted the data of patients admitted for bacterial pneumonia treatment from the discharge records of 42 hospitals between 1 April 2014 and 31 December 2019. Inclusion criteria were as follows: (1) those undergoing an unscheduled admission for admission-necessitating diagnosis of bacterial pneumonia (ICD-10 codes, J13–J15) with prognostic factors for pneumonia recorded, (2) those with an estimated glomerular filtration rate (eGFR) of < 60 ml/min/1.73 m^2^ on admission; and (3) age ≥ 60 years.

Exclusion criteria were as follows: (1) patients undergoing either of the following kidney replacement therapies within 2 days of admission: haemodialysis, peritoneal dialysis, or continuous haemodiafiltration, and (2) patients with missing BMI values or smoking history.

### Study variables

We extracted data related to the following variables: age; sex; BMI; Charlson comorbidity index; prognostic factors for pneumonia (presence of dehydration, respiratory failure, orientation disturbance, pulmonary consolidation, and hypotension); and eGFR calculated using age, sex, and serum creatinine based on the Japanese Nephrology Society equation [22]. When the laboratory data on the day of admission were absent, we used the corresponding value recorded the next day. We divided the BMI into four groups according to BMI quartiles: low, medium–low, medium–high, and high. The Charlson comorbidity index, which represents weighted comorbidities to predict in-hospital death [23], was calculated using the diagnoses present at the time of admission.

### Study outcomes

The primary outcome was in-hospital mortality. As a secondary outcome, we examined the length of hospital stay due to bacterial pneumonia among those who did not experience in-hospital death.

### Statistical analysis

We summarised the patient characteristics based on whether or not the patients underwent the primary outcome during hospitalisation. Patient characteristics were compared using the chi-square test for categorical variables and the Student’s t-test for continuous variables. Next, we compared the outcomes across the groups using the chi-square test for in-hospital mortality and analysis of variance for the length of stay.

To analyse the relationship between BMI and in-hospital mortality, we used generalised linear models with a logit link and a family of binomial distributions. For the secondary outcome, we used generalised linear models with an identity link and a family of Gaussian distributions and to examine the relationship between BMI and length of stay. The first model used the quadrisected BMI categories, and the second model used a restricted cubic spline with standard points of three knots (10^th^, 50^th^, and 90^th^ percentiles) with a BMI of 22 kg/m^2^ set as the reference because it is considered an “ideal BMI” among the Japanese population [24]. Likewise, those with a medium–high BMI were considered as a reference because this BMI category included the value of 22 kg/m^2^. In both models, we adjusted for the following potential confounders as independent variables: age, sex, CKD stages G3–G5, Charlson comorbidity index, and the aforementioned prognostic factors for pneumonia.

To examine the interaction between kidney function and BMI for in-hospital mortality or length of stay, we performed the following analyses: (1) treating both BMI and eGFR as nonlinear continuous variables and (2) evaluating the association between BMI and prognosis or length of stay stratified by CKD stages G3-5. The former analysis was performed to visualise the interaction between eGFR and BMI using a generalised additive model to describe the interaction between eGFR and BMI, which was made possible using mgcv::gam in R as reported previously [25]. We set those with an eGFR of 45 ml/min/1.73 m^2^ and a BMI of 22 kg/m^2^ as the reference.

We conducted four sensitivity analyses of the primary outcome. First, we used eGFR as a linear continuous variable instead of the CKD categories. Second, we excluded those with a length of stay ≤ 2 days. Third, we used multiple imputation on missing values on BMI and smoking history. Finally, we excluded patients complicated by acute kidney injury on admission.

All hypothetical tests had a two-sided significance level of 0.05, and all statistical analyses were conducted using Stata version 17 (StataCorp, College Station, TX, USA) and the statistical programming language R (version 3.6.1; R Foundation for Statistical Computing, Vienna, Austria).

## Results

### Study population

**Fig. 1:**
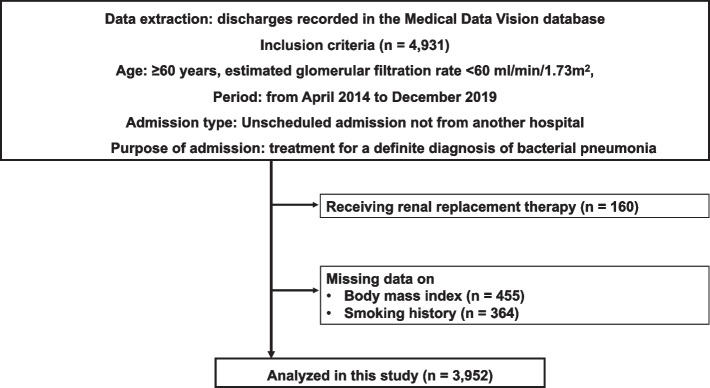
Flowchart of the patient selection process

Among the 4,931 patients who met the inclusion criteria, we excluded 979 patients who received kidney replacement therapy within 2 days of admission or those with missing data of BMI or smoking history (Fig. 1). We obtained data from 3,952 patients aged ≥ 60 years who had an unscheduled admission for pneumonia treatment, with 350 patients experiencing in-hospital death during hospitalisation.

Table 1 presents the background characteristics of the patients, stratified by whether or not they had in-hospital death. When compared with those not experiencing in-hospital death, patients who underwent in-hospital death were likely to be older, and have lower BMI, lower eGFR, and worse prognostic factors of pneumonia and Charlson comorbidity index.

**Table 1:** Characteristics of eligible patients categorised by the occurrence of in-hospital death

Variable	Category	Total	Not experiencing in-hospital death	Experiencing in-hospital death	*P* value
		*n* = 3,952	*n* = 3,602	*n* = 350	
Age (years)		83.1 (8.7)	82.9 (8.7)	85.7 (7.6)	< 0.001
Male		1,732 (43.8%)	1,575 (43.7%)	157 (44.9%)	0.68
BMI (kg/m^2^)		21.3 (4.0)	21.5 (4.0)	19.8 (4.0)	< 0.001
BMI category	Low, ≤ 18.5 kg/m^2^	987 (25.0%)	841 (23.3%)	146 (41.7%)	< 0.001
	Medium–low, 18.6–21.0 kg/m^2^	989 (25.0%)	912 (25.3%)	77 (22.0%)	
	Medium–high, 21.1–23.7 kg/m^2^	986 (24.9%)	918 (25.5%)	68 (19.4%)	
	High, ≥ 23.8 kg/m^2^	990 (25.1%)	931 (25.8%)	59 (16.9%)	
BMI category in Japan	Lean, < 18.5 kg/m^2^	969 (24.5%)	825 (22.9%)	144 (41.1%)	< 0.001
	Normal, 18.5–24.9 kg/m^2^	2,329 (58.9%)	2,158 (59.9%)	171 (48.9%)	
	Obese, ≥ 25.0 kg/m^2^	654 (16.5%)	619 (17.2%)	35 (10.0%)	
Smoking history	Non-smoker	2,440 (61.7%)	2,205 (61.2%)	235 (67.1%)	0.029
	Current/past smoker	1,512 (38.3%)	1,397 (38.8%)	115 (32.9%)	
Estimated glomerular filtration rate (ml/min/1.73 m^2^)		40.6 (13.6)	41.0 (13.3)	36.6 (15.7)	< 0.001
CKD staging	CKD stage G3	3,054 (77.3%)	2,829 (78.5%)	225 (64.3%)	< 0.001
	CKD stage G4	674 (17.1%)	589 (16.4%)	85 (24.3%)	
	CKD stage G5	224 (5.7%)	184 (5.1%)	40 (11.4%)	
Creatinine clearance (ml/min)		31.8 (14.3)	32.5 (14.3)	25.0 (12.2)	< 0.001
Dehydration		2,414 (61.1%)	2,129 (59.1%)	285 (81.4%)	< 0.001
Respiratory failure	None	2,129 (53.9%)	2,030 (56.4%)	99 (28.3%)	< 0.001
	Moderate	1,301 (32.9%)	1,181 (32.8%)	120 (34.3%)	
	Severe	522 (13.2%)	391 (10.9%)	131 (37.4%)	
Orientation disturbance		648 (16.4%)	495 (13.7%)	153 (43.7%)	< 0.001
Immunosuppression		664 (16.8%)	588 (16.3%)	76 (21.7%)	0.010
Pulmonary consolidation		1,033 (26.1%)	882 (24.5%)	151 (43.1%)	< 0.001
Hypotension on admission		288 (7.3%)	223 (6.2%)	65 (18.6%)	< 0.001
Pneumonia type	Community-acquired	3,724 (94.2%)	3,412 (94.7%)	312 (89.1%)	< 0.001
	Nursing and healthcare-associated	228 (5.8%)	190 (5.3%)	38 (10.9%)	
Charlson comorbidity index		1.7 (1.7)	1.6 (1.6)	2.0 (1.9)	< 0.001

Data are presented as mean (standard deviation) for continuous measures and as n (%) for categorical measures

*BMI* Body mass index, *CKD* Chronic kidney disease

Overall, 3,952 eligible patients were classified into the following groups: low, medium–low, medium–high, and high BMI. The cut-off points were 18.5, 21.0, and 23.7 kg/m^2^ based on the quartiles of BMI. Table 2 presents the patients’ background characteristics in each group. As BMI increased, patients tended to be younger and to have fewer prognostic factors (presence of dehydration, respiratory failure, orientation disturbance, pulmonary consolidation, or hypotension), whereas eGFR did not seem to differ. Table 3 summarises the unadjusted outcomes of the BMI groups, which showed that as BMI decreased, in-hospital mortality and length of stay increased.

**Table 2:** Characteristics of eligible patients categorised by quadrisected body mass index categories

Variable	Category	Quadrisected groups based on quartiles of body mass index				*P* value
		Low, ≤ 18.5 kg/m^2^	Medium–low, 18.6–21.0 kg/m^2^	Medium–high, 21.1–23.7 kg/m^2^	High, ≥ 23.8 kg/m^2^	
		*N* = 987	*N* = 989	*N* = 986	*N* = 990	
Age (years)		85.0 (8.4)	84.3 (8.6)	82.3 (8.6)	81.0 (8.5)	< 0.001
Male		482 (48.8%)	470 (47.5%)	371 (37.6%)	409 (41.3%)	< 0.001
Smoking history	Non-smoker	639 (64.7%)	635 (64.2%)	580 (58.8%)	586 (59.2%)	0.006
	Current/past smoker	348 (35.3%)	354 (35.8%)	406 (41.2%)	404 (40.8%)	
Estimated glomerular filtration rate (ml/min/1.73 m^2^)		40.6 (13.7)	40.2 (14.0)	41.1 (13.4)	40.3 (13.4)	0.49
CKD staging	CKD stage G3	766 (77.6%)	739 (74.7%)	780 (79.1%)	769 (77.7%)	0.39
	CKD stage G4	162 (16.4%)	191 (19.3%)	155 (15.7%)	166 (16.8%)	
	CKD stage G5	59 (6.0%)	59 (6.0%)	51 (5.2%)	55 (5.6%)	
Creatinine clearance (ml/min)		24.3 (9.6)	29.0 (12.0)	33.7 (13.0)	40.4 (16.6)	< 0.001
Dehydration		677 (68.6%)	624 (63.1%)	578 (58.6%)	535 (54.0%)	< 0.001
Respiratory failure	None	513 (52.0%)	525 (53.1%)	562 (57.0%)	529 (53.4%)	0.018
	Moderate	313 (31.7%)	336 (34.0%)	317 (32.2%)	335 (33.8%)	
	Severe	161 (16.3%)	128 (12.9%)	107 (10.9%)	126 (12.7%)	
Orientation disturbance		212 (21.5%)	184 (18.6%)	126 (12.8%)	126 (12.7%)	< 0.001
Immunosuppression		154 (15.6%)	171 (17.3%)	171 (17.3%)	168 (17.0%)	0.70
Pulmonary consolidation		245 (24.8%)	266 (26.9%)	268 (27.2%)	254 (25.7%)	0.60
Hypotension on admission		111 (11.2%)	79 (8.0%)	51 (5.2%)	47 (4.7%)	< 0.001
Pneumonia type	Community-acquired	900 (91.2%)	924 (93.4%)	947 (96.0%)	953 (96.3%)	< 0.001
	Nursing and healthcare-associated	87 (8.8%)	65 (6.6%)	39 (4.0%)	37 (3.7%)	
Charlson comorbidity index		1.7 (1.7)	1.7 (1.6)	1.7 (1.7)	1.6 (1.6)	0.57

Data are presented as mean (standard deviation) for continuous measures and as n (%) for categorical measures

*CKD* Chronic kidney disease

**Table 3:** Cross-tabulation of body mass index and outcomes

Outcome	Low, ≤ 18.5 kg/m^2^	Medium–low, 18.6–21.0 kg/m^2^	Medium–high, 21.1–23.7 kg/m^2^	High, ≥ 23.8 kg/m^2^	*P* value
In-hospital mortality	146 (14.8%)	77 (7.8%)	68 (6.9%)	59 (6.0%)	< 0.001
Length of stay	23.8 (22.0)	21.0 (19.5)	17.9 (17.0)	17.5 (16.0)	< 0.001

Binary and continuous measures are presented as n (%) and mean (standard deviation), respectively, and were tested using the chi-square test and analysis of variance, respectively. The length of stay was calculated for patients in whom in-hospital death did not occur

### Association between BMI and in-hospital mortality or length of stay

**Fig. 2:**
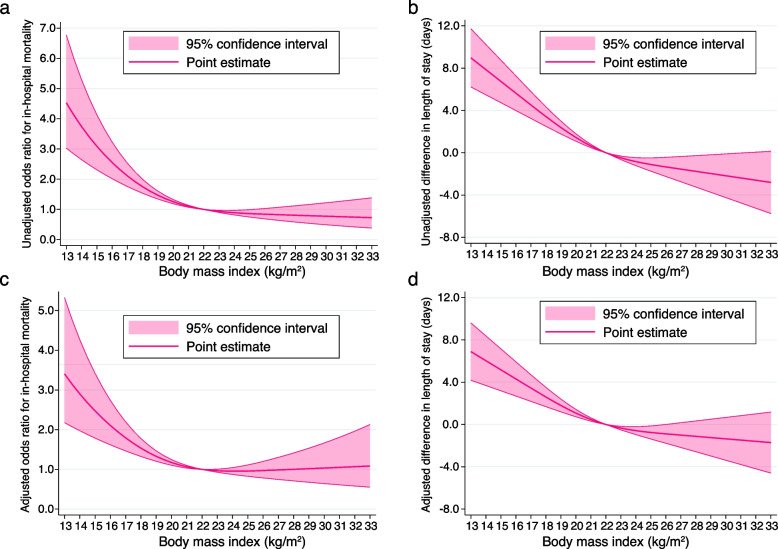
Cubic spline showing association of body mass index, with in-hospital death and length of stay. **a** Cubic spline showing association between the body mass index and in-hospital death as odds ratio in the unadjusted model. **b** Cubic spline showing the association between the body mass index and length of stay as the difference (days) in the unadjusted model. **c** Cubic spline showing the association between the body mass index and in-hospital death as odds ratio in the adjusted model. **d** Cubic spline showing the association between the body mass index and length of stay as the difference (days) in the adjusted model

The unadjusted results of the primary and secondary outcomes using a restricted cubic spline model in relation to BMI change are shown in Fig. 2a and b. The curves were L-shaped and when compared with patients with a BMI of 22.0 kg/m^2^, mortality and length of stay were higher and longer in those with a BMI of < 22.0 kg/m^2^, respectively, while patients with a BMI of > 22.0 kg/m^2^ did not yield this result.

The results of the analyses, performed using a categorical model adjusted for background characteristics, are presented in Table 4. The multivariable adjustment did not alter the association between BMI and in-hospital mortality or length of hospital stay; the coefficients of the other variables are shown in Supplementary Table 1 (see Additional File 1).

**Table 4:** The outcomes based on body mass index categories in multivariable regression analyses

Type of outcome	In-hospital mortality					Length of stay				
Body mass index	Odds ratio	95% Confidence interval			*P* value	Difference	95% Confidence interval			*P* value
Low, ≤ 18.5 kg/m^2^	1.74	1.25	-	2.41	0.001	4.43	2.71	-	6.15	< 0.001
Medium–low, 18.6–21.0 kg/m^2^	0.87	0.60	-	1.24	0.43	2.01	0.34	-	3.68	0.018
Medium–high, 21.1–23.7 kg/m^2^	Reference					Reference				
High, ≥ 23.8 kg/m^2^	0.86	0.58	-	1.25	0.43	-0.27	-1.93	-	1.38	0.75

The length of stay was calculated for patients in whom in-hospital death did not occur

The adjusted results of the primary and secondary outcomes using a restricted cubic spline model are shown in Fig. 2c and d, respectively. These results were consistent with those of the analyses using BMI as a nonlinear continuous variable. The curve showing the association between BMI and in-hospital mortality was L-shaped. The other coefficients are shown in Supplementary Table 2 (see Additional File 2). Compared with patients with a BMI of 22.0 kg/m^2^, those with a BMI of 18.5 kg/m^2^ had a higher risk of death (odds ratio: 1.43; 95% confidence interval:1.26 to 1.61), and this risk increased monotonically as BMI decreased from 22.0 kg/m^2^. However, this trend was not observed in patients with a BMI > 22.0 kg/m^2^.

### Interaction between kidney function and BMI for outcomes

**Fig. 3:**
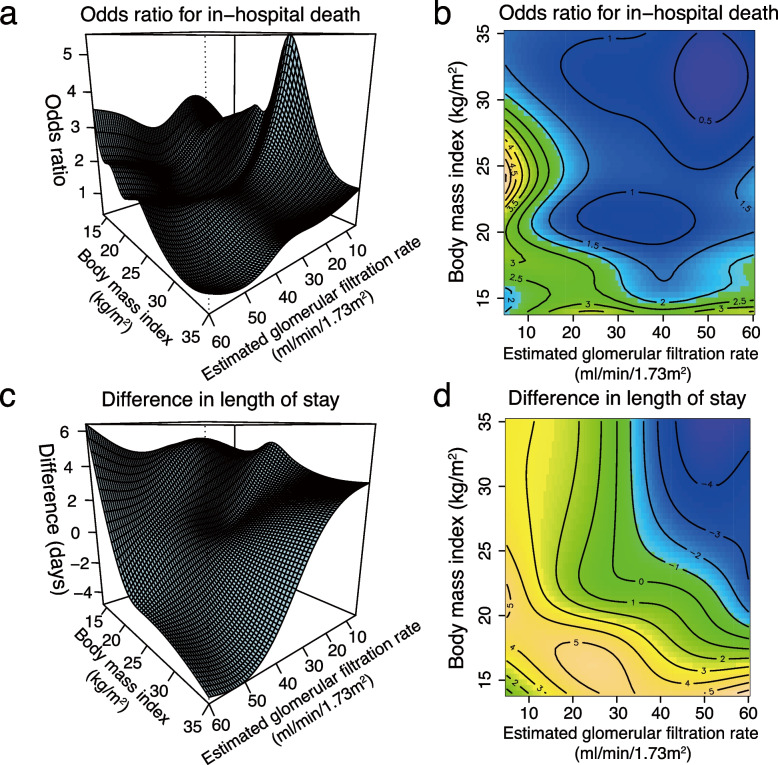
Three-dimensional graph showing the association of body mass index with in-hospital death and the length of stay.The figure concurrently shows their interactive effect in the multivariable model with the reference of an estimated glomerular rate of 45 ml/min/1.73 m^2^, and a body mass index of 22 kg/m^2^. **a** Three-dimensional plot of the odds ratio for in-hospital mortality. **b** Contour plot of the odds ratio for in-hospital mortality. The solid black curve indicates the same odds ratio. **c** Three-dimensional plot of the coefficients for differences in length of stay (days). **d** Contour plot of the coefficients for the difference in the length of stay (days). The solid black curve indicates the same coefficient

**Fig. 4:**
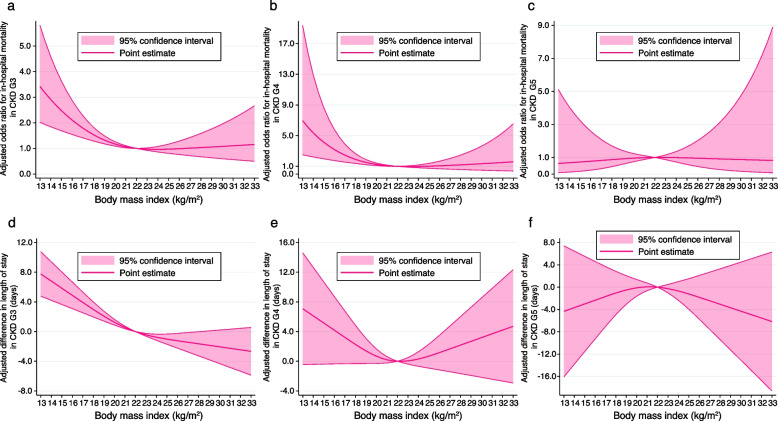
Cubic spline showing odds ratio or difference in length of stay stratified by kidney function. **a** Association between BMI and the risk of in-hospital mortality in patients with CKD stage G3 after multivariable adjustment. **b** Association between BMI and the risk of in-hospital mortality in patients with CKD stage G4 after multivariable adjustment. **c** Association between BMI and the risk of in-hospital mortality in patients with CKD stage G5 after multivariable adjustment. **d** Association between BMI and difference in length of stay (days) in patients with CKD stage G3 after multivariable adjustment. **e** Association between BMI and difference in length of stay (days) in patients with CKD stage G4 after multivariable adjustment. **f** Association between BMI and difference in the length of stay (days) in patients with CKD stage G5 after multivariable adjustment. CKD G3, chronic kidney disease stage G3; CKD G4, chronic kidney disease stage G4; CKD G5, chronic kidney disease stage G5

The generalised additive model examining the interaction between BMI and eGFR regarding the risk of death and difference in the length of hospital stay is shown in Fig. 3, and the other coefficients are shown in Supplementary Table 3 (see Additional File 3). When comparing the reference eGFR of 45 ml/min/1.73 m^2^ and reference BMI of 22 kg/m^2^, the decrease in either eGFR or BMI was generally associated with an increased risk of in-hospital death and longer length of stay. However, as eGFR decreased, the risk reduction in death or shorter hospital stay in parallel with BMI increase weakened. Figure 4 shows the results of the analyses stratified by CKD stages G3–5. As CKD stages advanced, the association between BMI < 22 kg/m^2^ and an increased risk of death or length of hospital stay became less conspicuous. For example, the association between BMI and in-hospital mortality was L-shaped in those with CKD stages G3–4, as shown in Fig. 4a and b, while that in those with CKD stage G5 was not, as shown in Fig. 4c; the association between BMI and length of stay was L-shaped in those with CKD stage G3, as shown in Fig. 4d, while that in those with CKD stages G4–5 was not, as shown in Fig. 4e and f.

### Sensitivity analysis

**Fig. 5:**
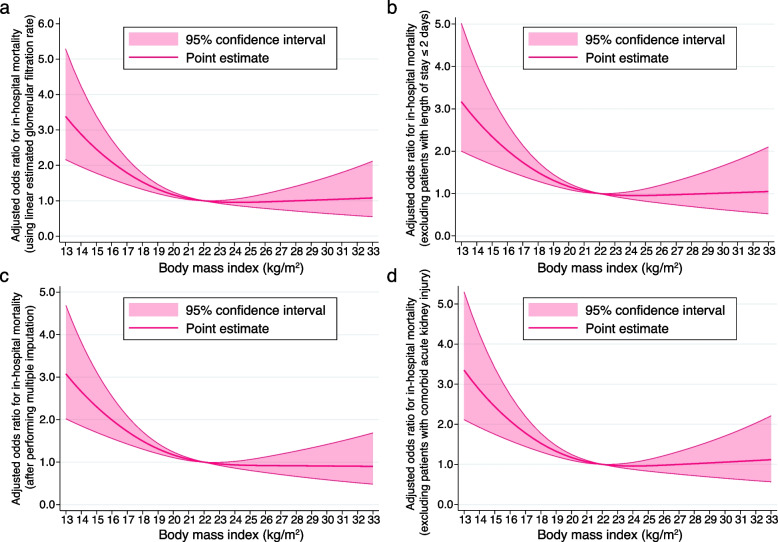
Cubic spline showing the association between body mass index and in-hospital death in the sensitivity analyses. **a** Sensitivity analysis 1: treating the estimated glomerular filtration rate (eGFR) as a linear continuous variable in the multivariable-adjusted model. **b** Sensitivity analysis 2: multivariable-adjusted analysis after excluding those with the length of stay of ≤ 2 days. **c** Sensitivity analysis 3: multivariable-adjusted analysis using multiple imputations for missing values of smoking history and body mass index. **d** Sensitivity analysis 4: multivariable-adjusted analysis performed after excluding those complicated with acute kidney injury on admission

We analysed data from 3,952, 3,913, 4,771, and 3,802 patients in sensitivity analyses 1, 2, 3, and 4, respectively. The associations between BMI and in-hospital mortality in the sensitivity analyses were similar to those obtained in the main analysis (Table 5). Figure 5 demonstrates the cubic spline curves for the sensitivity analyses, and details of the other coefficients are presented in Supplementary Tables 4–7 (see Additional files 4, 5, 6, and 7).

**Table 5:** Odds ratios for in-hospital mortality by body mass index categories in sensitivity analyses

Type of analysis	Sensitivity analysis 1 (continuous linear eGFR)					Sensitivity analysis 2 (limiting to length of stay > 2)				
Body mass index	Odds ratio	95% CI			*P* value	Odds ratio	95% CI			*P* value
Low	1.73	1.25	-	2.40	0.001	1.50	1.08	-	2.10	0.017
Medium–low	0.87	0.61	-	1.25	0.45	0.81	0.56	-	1.16	0.26
Medium–high	Reference					Reference				
High	0.86	0.59	-	1.26	0.44	0.94	0.64	-	1.38	0.74
Type of analysis	Sensitivity analysis 3 (multiple imputation)					Sensitivity analysis 4 (excluding those with AKI)				
Body mass index	Odds ratio	95% CI			*P* value	Odds ratio	95% CI			*P* value
Low	1.79	1.31	-	2.45	< 0.001	1.69	1.21	-	2.36	< 0.001
Medium–low	0.97	0.68	-	1.37	0.85	0.88	0.61	-	1.27	0.49
Medium–high	Reference					Reference				
High	0.88	0.61	-	1.27	0.50	0.87	0.59	-	1.29	0.49

*eGFR* Estimated glomerular filtration rate, *OR* Odds ratio, *CI* Confidence interval, *AKI* Acute kidney injury

## Discussion

In the present observational study conducted using multicentre admission data in Japan, we observed that a lower BMI was associated with poor prognosis in older patients with CKD without dialysis dependency. This study highlights the association between low BMI and in-hospital mortality in older individuals with CKD admitted for bacterial pneumonia after adjusting for background characteristics.

In-hospital mortality in this study (8.9%) was higher than that in a previous study (2.2%) targeting community-acquired pneumonia in a younger population [26] and lower than the 30-day mortality after admission for pneumonia in a similarly aged population (12.1%) [27]. Deaths related to pneumonia admission were reportedly associated with advanced age and comorbidities [26], which is consistent with the data of this study in that the risk factors for death included advanced age, a higher number of comorbidities, and advanced CKD stages.

Even after adjusting for CKD stages, pre-existing comorbidities, and advanced age, a lower BMI was associated with higher mortality in our study. The results of our study were compatible with the association of BMI with mortality as an L-shaped curve among patients undergoing regular dialysis [8]. Although the exact mechanism remains unclear in the present study, there are possible explanations for the association between low BMI and mortality. Low BMI is deeply connected to frailty in older people; hence, presumably, those with low BMI may have been vulnerable to infection [28]. This assumption is supported by the fact that frailty is an independent prognostic factor in older patients with acute myocardial infarction, heart failure, and pneumonia [29]. Another explanation could be the association of low BMI with factors related to poor prognoses, like sarcopenia [30], malnutrition [31], and difficulty in swallowing [32]. These factors influence each other [33] and therefore, comprehensive approaches against them are warranted.

We revealed an interaction between kidney function and BMI for in-hospital mortality using stratification and visualisation (Figs. 3 and 4). Evidence on risk stratification by CKD stages G3-5 was previously insufficient in terms of in-hospital mortality; only two analyses stratified by eGFR of 30 or 45 ml/min/1.73 m^2^ were reported [34, 35]. We found a nonlinear interaction between eGFR and BMI; as eGFR decreased, the protective effect of BMI increase was diminished. This interaction was similar to that reported in a previous study on the interaction for mortality within two years after myocardial infarction among patients with non-dialysis-dependent CKD, which showed that as kidney function decreased, the protective association between high BMI and death after myocardial infarction also weakened [16]. This was probably because the effects of eGFR decrease on mortality were presumably stronger than those of high BMI, as previously considered [16]. Further studies are warranted to confirm the interactive effects of advanced CKD stages and their pathophysiology.

The strengths of our study exist mainly in our observation that we focused on a non-dialysis-dependent CKD population stratified by the extent of advanced CKD stages and revealed a nonlinear interaction between BMI and kidney function. As mentioned above, only a few previous studies have examined the association between a low BMI and mortality in such a population, while most of them focused on in-hospital mortality among people with dialysis dependency. Our observation was made possible probably because Japan has the highest degree of ageing [36] and a high prevalence of CKD among Asian countries [37]. We also succeeded in demonstrating a nonlinear interaction between BMI and kidney function; however, no previous studies have clarified this association.

This study has several limitations. First, because of the nature of the database, we could not confirm whether all individuals in the included population had the same eGFR before admission because the pre-admission eGFR was not necessarily available. However, if we had limited the analysis population to patients with an available eGFR before admission, in other words, patients who had a history of examination at the hospital before admission, it could have introduced selection bias. However, even after excluding those with comorbid acute kidney injury on admission (*n* = 150), the results did not change. Thus, our results are robust. Second, given the nature of the cohort study, there might be residual unmeasured confounders. For example, we did not adjust for proteinuria severity or nutritional markers, like prealbumin, due to the lack of corresponding data. Other potential unmeasured confounders included psychosocial-related, sarcopenia-related, or factors associated with the swallowing function. These factors are linked to mortality [38, 39]; therefore, future studies are needed to adjust for them. Third, we did not have information on mortality after discharge due to the nature of the dataset; therefore, we could not perform a survival analysis. Finally, the mean age and BMI were 83.1 years and 21.3 kg/m^2^, respectively, in the population analysed in our study, while a previous study among those on regular dialysis used a cohort with a mean age of 61.0 years and a mean BMI of 26.8 kg/m^2^ [8], and therefore, our results may not be generalisable to a younger population and those with advanced obesity.

## Conclusion

This retrospective cohort study, conducted using a large-scale nationwide claims database, revealed that lower BMI may be a risk factor for death due to bacterial pneumonia and that the protective effect of high BMI may weaken as eGFR decreases in older people with non-dialysis-dependent CKD. This finding suggests that healthcare providers and policymakers should take steps to prevent low BMI in older people with CKD.

## Supplementary Information

**Additional file 1:** **Table 1.** Odds ratios for in-hospital mortality and coefficients for the length of stay for covariates estimated using the multivariable regression analysis (using body mass index as a categorical variable).

**Additional file 2:** **Table 2.** Odds ratios for in-hospital mortality and coefficients for the length of stay, estimated using the multivariable regression analysis (using body mass index as a non-linear continuous variable).

**Additional file 3:** **Table 3.** Odds ratios for in-hospital mortality and coefficients for the length of stay, estimated using the multivariable regression analysis (using a generalised additive model).

**Additional file 4:** **Table 4.** Odds ratios for in-hospital mortality estimated using the multivariable regression analysis (sensitivity analysis 1).

**Additional file 5:** **Table 5.** Odds ratios for in-hospital mortality for covariates in the multivariable regression analysis (sensitivity analysis 2).

**Additional file 6:** **Table 6.** Odds ratios for in-hospital mortality for covariates in the multivariable regression analysis (sensitivity analysis 3).

**Additional file 7:** **Table 7.** Odds ratios for in-hospital mortality for covariates in the multivariable regression analysis (sensitivity analysis 4).

## Data Availability

The data that support the findings of this study are available from Medical Data Vision Co., Ltd. (Tokyo, Japan) but restrictions apply to the availability of these data, which were used under license for the current study, and so are not publicly available. Data are however available from the corresponding author upon reasonable request and with permission of Medical Data Vision Co., Ltd.

## Abbreviations

BMI: Body mass index
CKD: Chronic kidney disease
MDV: Medical Data Vision
DPC: Diagnosis and procedure combination
eGFR: Estimated glomerular filtration rate
