# Supplementary material for: Impact of body mass index on in-hospital mortality in older patients hospitalized for bacterial pneumonia with non-dialysis-dependent chronic kidney disease
Source: BMC Geriatr. 2022 Dec 9;22:950. doi: 10.1186/s12877-022-03659-3 (PMC9733221; doi:10.1186/s12877-022-03659-3)
Supplement: Supplementary file 1 — Additional file 1: Table 1. Odds ratios for in-hospital mortality and coefficients for the length of stay for covariates estimated using the multivariable regression analysis (using body mass index as a categorical variable). [file 12877_2022_3659_MOESM1_ESM.docx]

**Supplementary Table 1. Odds ratios for in-hospital mortality and coefficients for the length of stay for covariates estimated using the multivariable regression analysis (using body mass index as a categorical variable).**

| Outcome | | In-hospital mortality | | | | | Length of stay | | | | |
| --- | --- | --- | --- | --- | --- | --- | --- | --- | --- | --- | --- |
| Variable | Category | Odds ratio | 95% Confidence interval | | | P value | Difference | 95% Confidence interval | | | P value |
| Age (10-year increase) | | 1.43 | 1.21 | - | 1.68 | <0.001 | 1.43 | 0.69 | - | 2.16 | <0.001 |
| Sex | Female | Reference | |  |  |  | Reference | |  |  |  |
|  | Male | 0.82 | 0.61 | - | 1.09 | 0.17 | -0.19 | -1.63 | - | 1.25 | 0.80 |
| CKD stage | G3 | Reference | |  |  |  | Reference | |  |  |  |
|  | G4 | 1.32 | 0.98 | - | 1.77 | 0.065 | 2.83 | 1.17 | - | 4.49 | 0.001 |
|  | G5 | 1.93 | 1.27 | - | 2.94 | 0.002 | 2.27 | -0.47 | - | 5.01 | 0.104 |
| Smoking status | Non-smoker | Reference |  |  |  |  | Reference |  |  |  |  |
|  | Current/past smoker | 0.77 | 0.57 | - | 1.04 | 0.083 | -1.81 | -3.27 | - | -0.34 | 0.016 |
| Dehydration | | 1.59 | 1.17 | - | 2.16 | 0.003 | 2.51 | 1.22 | - | 3.80 | <0.001 |
| Respiratory failure | None | Reference | |  |  |  | Reference | |  |  |  |
|  | Moderate | 1.76 | 1.32 | - | 2.34 | <0.001 | 2.17 | 0.85 | - | 3.49 | 0.001 |
|  | Severe | 3.61 | 2.62 | - | 4.98 | <0.001 | 7.26 | 5.20 | - | 9.32 | <0.001 |
| Orientation disturbance | | 2.73 | 2.10 | - | 3.55 | <0.001 | 6.21 | 4.43 | - | 7.99 | <0.001 |
| Immunosuppression | | 1.52 | 1.11 | - | 2.08 | 0.009 | -1.41 | -3.09 | - | 0.27 | 0.10 |
| Pulmonary consolidation | | 1.53 | 1.18 | - | 1.99 | 0.001 | 1.49 | 0.06 | - | 2.92 | 0.041 |
| Hypotension | | 1.52 | 1.07 | - | 2.17 | 0.020 | 0.55 | -1.96 | - | 3.07 | 0.67 |
| Pneumonia type | Community-acquired | Reference | |  |  |  | Reference | |  |  |  |
|  | Nursing and healthcare-associated | 1.50 | 1.00 | - | 2.26 | 0.051 | 1.98 | -0.70 | - | 4.67 | 0.15 |
| Charlson comorbidity index | | 1.11 | 1.03 | - | 1.19 | 0.004 | 0.61 | 0.23 | - | 0.99 | 0.002 |

CKD, chronic kidney disease.

This multivariable regression analysis was performed by considering body mass index as a categorical variable. Length of stay is summarized/calculated for those in whom in-hospital death did not occur.
