# Supplementary material for: Impact of body mass index on in-hospital mortality in older patients hospitalized for bacterial pneumonia with non-dialysis-dependent chronic kidney disease
Source: BMC Geriatr. 2022 Dec 9;22:950. doi: 10.1186/s12877-022-03659-3 (PMC9733221; doi:10.1186/s12877-022-03659-3)
Supplement: Supplementary file 2 — Additional file 2: Table 2. Odds ratios for in-hospital mortality and coefficients for the length of stay, estimated using the multivariable regression analysis (using body mass index as a non-linear continuous variable). [file 12877_2022_3659_MOESM2_ESM.docx]

**Supplementary Table 2. Odds ratios for in-hospital mortality and coefficients for the length of stay, estimated using the multivariable regression analysis (using body mass index as a non-linear continuous variable).**

| Outcome | | In-hospital mortality | | | | | Length of stay | | | | |
| --- | --- | --- | --- | --- | --- | --- | --- | --- | --- | --- | --- |
| Variable | Category | Odds ratio | 95% Confidence interval | | | P value | Difference | 95% Confidence interval | | | P value |
| Age (10-year increase) | | 1.44 | 1.22 | - | 1.69 | <0.001 | 1.42 | 0.69 | - | 2.16 | <0.001 |
| Sex | Female | Reference | |  |  |  | Reference | |  |  |  |
|  | Male | 0.80 | 0.60 | - | 1.06 | 0.12 | -0.17 | -1.61 | - | 1.27 | 0.82 |
| CKD stage | G3 | Reference | |  |  |  | Reference | |  |  |  |
|  | G4 | 1.32 | 0.98 | - | 1.77 | 0.065 | 2.86 | 1.20 | - | 4.52 | 0.001 |
|  | G5 | 1.96 | 1.29 | - | 2.98 | 0.002 | 2.25 | -0.48 | - | 4.99 | 0.107 |
| Smoking status | Non-smoker | Reference |  |  |  |  | Reference |  |  |  |  |
|  | Current/past smoker | 0.77 | 0.57 | - | 1.04 | 0.094 | -1.83 | -3.29 | - | -0.37 | 0.014 |
| Dehydration | | 1.57 | 1.16 | - | 2.13 | 0.004 | 2.49 | 1.20 | - | 3.79 | <0.001 |
| Respiratory failure | None | Reference | |  |  |  | Reference | |  |  |  |
|  | Moderate | 1.75 | 1.31 | - | 2.34 | <0.001 | 2.21 | 0.89 | - | 3.53 | 0.001 |
|  | Severe | 3.60 | 2.61 | - | 4.96 | <0.001 | 7.31 | 5.26 | - | 9.37 | <0.001 |
| Orientation disturbance | | 2.65 | 2.04 | - | 3.45 | <0.001 | 6.15 | 4.38 | - | 7.93 | <0.001 |
| Immunosuppression | | 1.52 | 1.11 | - | 2.08 | 0.009 | -1.42 | -3.10 | - | 0.26 | 0.097 |
| Pulmonary consolidation | | 1.54 | 1.19 | - | 1.99 | 0.001 | 1.48 | 0.05 | - | 2.91 | 0.042 |
| Hypotension | | 1.50 | 1.05 | - | 2.13 | 0.026 | 0.46 | -2.06 | - | 2.97 | 0.72 |
| Pneumonia type | Community-acquired | Reference | |  |  |  | Reference | |  |  |  |
|  | Nursing and healthcare-associated | 1.48 | 0.99 | - | 2.23 | 0.058 | 1.94 | -0.74 | - | 4.63 | 0.16 |
| Charlson comorbidity index | | 1.11 | 1.04 | - | 1.19 | 0.003 | 0.61 | 0.23 | - | 0.99 | 0.002 |

CKD, chronic kidney disease.

This multivariable regression analysis was performed by considering body mass index as a nonlinear continuous variable. Length of stay is summarized/calculated for those in whom in-hospital death did not occur.
