# Supplementary material for: Impact of body mass index on in-hospital mortality in older patients hospitalized for bacterial pneumonia with non-dialysis-dependent chronic kidney disease
Source: BMC Geriatr. 2022 Dec 9;22:950. doi: 10.1186/s12877-022-03659-3 (PMC9733221; doi:10.1186/s12877-022-03659-3)
Supplement: Supplementary file 3 — Additional file 3: Table 3. Odds ratios for in-hospital mortality and coefficients for the length of stay, estimated using the multivariable regression analysis (using a generalised additive model). [file 12877_2022_3659_MOESM3_ESM.docx]

**Supplementary Table 3. Odds ratios for in-hospital mortality and coefficients for the length of stay, estimated using the multivariable regression analysis (using a generalised additive model).**

| Outcome | | In-hospital mortality | | | | | Length of stay | | | | |
| --- | --- | --- | --- | --- | --- | --- | --- | --- | --- | --- | --- |
| Variable | Category | Odds ratio | 95% Confidence interval | | | P value | Difference | 95% Confidence interval | | | P value |
| Age (10-year increase) | | 1.49 | 1.26 | - | 1.76 | <0.001 | 1.37 | 0.64 | - | 2.10 | 0.064 |
| Sex | Female | Ref |  |  |  |  | Ref |  |  |  |  |
|  | Male | 0.81 | 0.61 | - | 1.09 | 0.16 | -0.21 | -1.65 | - | 1.23 | <0.001 |
| Smoking status | Non-smoker | Reference |  |  |  |  | Reference |  |  |  |  |
|  | Current/past smoker | 0.78 | 0.58 | - | 1.06 | 0.108 | -1.84 | -3.30 | - | -0.38 | 0.003 |
| Dehydration | | 1.65 | 1.21 | - | 2.27 | 0.002 | 2.06 | 0.72 | - | 3.39 | 0.69 |
| Respiratory failure | None | Ref |  |  |  |  | Ref |  |  |  |  |
|  | Moderate | 1.76 | 1.32 | - | 2.36 | <0.001 | 2.25 | 0.93 | - | 3.57 | 0.003 |
|  | Severe | 3.58 | 2.59 | - | 4.95 | <0.001 | 7.24 | 5.18 | - | 9.29 | <0.001 |
| Orientation disturbance | | 2.69 | 2.06 | - | 3.51 | <0.001 | 6.25 | 4.47 | - | 8.03 | <0.001 |
| Immunosuppression | | 1.55 | 1.13 | - | 2.13 | 0.007 | -1.39 | -3.07 | - | 0.29 | <0.001 |
| Pulmonary consolidation | | 1.53 | 1.18 | - | 1.99 | 0.001 | 1.42 | -0.01 | - | 2.85 | 0.10 |
| Hypotension | | 1.56 | 1.09 | - | 2.23 | 0.014 | 0.51 | -2.00 | - | 3.02 | 0.014 |
| Pneumonia type | Community-acquired | Reference |  |  |  |  | Reference |  |  |  |  |
|  | Nursing and healthcare-associated | 1.54 | 1.02 | - | 2.32 | 0.040 | 1.85 | -0.84 | - | 4.53 | 0.051 |
| Charlson comorbidity index | | 1.11 | 1.03 | - | 1.19 | 0.004 | 0.58 | 0.20 | - | 0.96 | 0.775 |

CKD, chronic kidney disease.

This multivariable regression analysis was performed by using a smooth spline, adjusting for interaction between body mass index and estimated glomerular filtration rate as a nonlinear continuous variable. Length of stay is summarized/calculated for those in whom in-hospital death did not occur.
