# Supplementary material for: Impact of body mass index on in-hospital mortality in older patients hospitalized for bacterial pneumonia with non-dialysis-dependent chronic kidney disease
Source: BMC Geriatr. 2022 Dec 9;22:950. doi: 10.1186/s12877-022-03659-3 (PMC9733221; doi:10.1186/s12877-022-03659-3)
Supplement: Supplementary file 4 — Additional file 4: Table 4. Odds ratios for in-hospital mortality estimated using the multivariable regression analysis (sensitivity analysis 1). [file 12877_2022_3659_MOESM4_ESM.docx]

**Supplementary Table 4. Odds ratios for in-hospital mortality estimated using the multivariable regression analysis (sensitivity analysis 1).**

| Variable | Category | Model treating body mass index as a categorical variable | | | | | Model treating body mass index as a nonlinear continuous variable | | | | |
| --- | --- | --- | --- | --- | --- | --- | --- | --- | --- | --- | --- |
|  |  | Odds ratio | 95% Confidence interval | | | P value | Odds ratio | 95% Confidence interval | | | P value |
| Age (10-year increase) | | 1.42 | 1.21 | - | 1.67 | <0.001 | 1.42 | 1.21 | - | 1.68 | <0.001 |
| Sex | Female | Reference |  |  |  |  | Reference |  |  |  |  |
|  | Male | 0.83 | 0.62 | - | 1.10 | 0.20 | 0.81 | 0.61 | - | 1.08 | 0.15 |
| Estimated glomerular filtration rate  (10 ml/min/1.73 m^2^ decrease) | | 0.92 | 0.84 | - | 1.00 | 0.058 | 0.91 | 0.83 | - | 1.00 | 0.043 |
| Smoking status | Non-smoker | Reference |  |  |  |  | Reference |  |  |  |  |
|  | Current/past smoker | 0.76 | 0.56 | - | 1.02 | <0.001 | 0.76 | 0.56 | - | 1.03 | 0.077 |
| Dehydration | | 1.60 | 1.17 | - | 2.18 | 0.003 | 1.57 | 1.15 | - | 2.14 | 0.004 |
| Respiratory failure | None | Reference |  |  |  |  | Reference |  |  |  |  |
|  | Moderate | 1.75 | 1.31 | - | 2.33 | <0.001 | 1.74 | 1.31 | - | 2.33 | <0.001 |
|  | Severe | 3.58 | 2.60 | - | 4.93 | <0.001 | 3.56 | 2.58 | - | 4.90 | <0.001 |
| Orientation disturbance | | 2.75 | 2.11 | - | 3.57 | <0.001 | 2.67 | 2.05 | - | 3.46 | <0.001 |
| Immunosuppression | | 1.52 | 1.11 | - | 2.08 | 0.009 | 1.52 | 1.11 | - | 2.08 | 0.009 |
| Pulmonary consolidation | | 1.56 | 1.20 | - | 2.01 | 0.001 | 1.56 | 1.21 | - | 2.02 | 0.001 |
| Hypotension | | 1.55 | 1.09 | - | 2.20 | 0.015 | 1.52 | 1.07 | - | 2.16 | 0.020 |
| Pneumonia type | Community-acquired | Reference |  |  |  |  | Reference |  |  |  |  |
|  | Nursing and healthcare-associated | 1.51 | 1.01 | - | 2.27 | 0.047 | 1.38 | 1.01 | - | 1.88 | 0.045 |
| Charlson comorbidity index | | 1.11 | 1.04 | - | 1.19 | 0.003 | 1.12 | 1.04 | - | 1.20 | 0.002 |

This multivariable regression analysis was performed by treating estimated glomerular filtration rate as a linear continuous variable. Length of stay is summarized/calculated for those in whom in-hospital death did not occur.
