# Supplementary material for: Impact of body mass index on in-hospital mortality in older patients hospitalized for bacterial pneumonia with non-dialysis-dependent chronic kidney disease
Source: BMC Geriatr. 2022 Dec 9;22:950. doi: 10.1186/s12877-022-03659-3 (PMC9733221; doi:10.1186/s12877-022-03659-3)
Supplement: Supplementary file 5 — Additional file 5: Table 5. Odds ratios for in-hospital mortality for covariates in the multivariable regression analysis (sensitivity analysis 2). [file 12877_2022_3659_MOESM5_ESM.docx]

**Supplementary Table 5. Odds ratios for in-hospital mortality for covariates in the multivariable regression analysis (sensitivity analysis 2).**

| Variable | Category | Model treating body mass index as a categorical variable | | | | | Model treating body mass index as a nonlinear continuous variable | | | | |
| --- | --- | --- | --- | --- | --- | --- | --- | --- | --- | --- | --- |
|  |  | Odds ratio | 95% Confidence interval | | | *P* value | Odds ratio | 95% Confidence interval | | | *P* value |
| Age (10-year increase) | | 1.42 | 1.20 | - | 1.67 | <0.001 | 1.42 | 1.20 | - | 1.68 | <0.001 |
| Sex | Female | Reference |  |  |  |  | Reference |  |  |  |  |
|  | Male | 0.79 | 0.59 | - | 1.06 | 0.12 | 0.77 | 0.58 | - | 1.04 | 0.084 |
| CKD stage | G3 | Reference |  |  |  |  |  |  |  |  |  |
|  | G4 | 1.25 | 0.93 | - | 1.70 | 0.15 | 1.25 | 0.93 | - | 1.70 | 0.14 |
|  | G5 | 1.97 | 1.29 | - | 3.00 | 0.002 | 1.99 | 1.31 | - | 3.03 | 0.001 |
| Smoking status | Non-smoker | Reference |  |  |  |  | Reference |  |  |  |  |
|  | Current/past smoker | 0.77 | 0.57 | - | 1.04 | 0.090 | 0.77 | 0.57 | - | 1.05 | 0.098 |
| Dehydration | | 1.55 | 1.14 | - | 2.10 | 0.005 | 1.53 | 1.12 | - | 2.08 | 0.007 |
| Respiratory failure | None | Reference |  |  |  |  | Reference |  |  |  |  |
|  | Moderate | 1.69 | 1.26 | - | 2.27 | <0.001 | 1.69 | 1.26 | - | 2.26 | <0.001 |
|  | Severe | 3.36 | 2.42 | - | 4.66 | <0.001 | 3.34 | 2.41 | - | 4.64 | <0.001 |
| Orientation disturbance | | 2.76 | 2.11 | - | 3.60 | <0.001 | 2.68 | 2.05 | - | 3.50 | <0.001 |
| Immunosuppression | | 1.50 | 1.09 | - | 2.06 | 0.013 | 1.49 | 1.08 | - | 2.05 | 0.014 |
| Pulmonary consolidation | | 1.60 | 1.23 | - | 2.08 | <0.001 | 1.60 | 1.23 | - | 2.09 | <0.001 |
| Hypotension | | 1.37 | 0.95 | - | 1.99 | 0.09 | 1.35 | 0.93 | - | 1.96 | 0.11 |
| Pneumonia type | Community-acquired | Reference |  |  |  |  | Reference |  |  |  |  |
|  | Nursing and healthcare-associated | 1.60 | 1.06 | - | 2.40 | 0.025 | 1.58 | 1.05 | - | 2.37 | 0.028 |
| Charlson comorbidity index | | 1.11 | 1.04 | - | 1.19 | 0.003 | 1.12 | 1.04 | - | 1.20 | 0.002 |

CKD, chronic kidney disease.

This multivariable regression analysis was performed, excluding those with a length of stay < 3 days. Length of stay is summarized/calculated for those in whom in-hospital death did not occur.
