# Supplementary material for: Impact of body mass index on in-hospital mortality in older patients hospitalized for bacterial pneumonia with non-dialysis-dependent chronic kidney disease
Source: BMC Geriatr. 2022 Dec 9;22:950. doi: 10.1186/s12877-022-03659-3 (PMC9733221; doi:10.1186/s12877-022-03659-3)
Supplement: Supplementary file 6 — Additional file 6: Table 6. Odds ratios for in-hospital mortality for covariates in the multivariable regression analysis (sensitivity analysis 3). [file 12877_2022_3659_MOESM6_ESM.docx]

**Supplementary Table 6. Odds ratios for in-hospital mortality for covariates in the multivariable regression analysis (sensitivity analysis 3).**

| Variable | Category | Model treating body mass index as a categorical variable | | | | | Model treating body mass index as a nonlinear continuous variable | | | | |
| --- | --- | --- | --- | --- | --- | --- | --- | --- | --- | --- | --- |
|  |  | Odds ratio | 95% Confidence interval | | | *P* value | Odds ratio | 95% Confidence interval | | | *P* value |
| Age (10-year increase) | | 1.53 | 1.33 | - | 1.77 | <0.001 | 1.53 | 1.33 | - | 1.77 | <0.001 |
| Sex | Female | Reference |  |  |  |  | Reference |  |  |  |  |
|  | Male | 0.81 | 0.63 | - | 1.05 | 0.11 | 0.80 | 0.62 | - | 1.03 | 0.084 |
| CKD stage | G3 | Reference |  |  |  |  |  |  |  |  |  |
|  | G4 | 1.44 | 1.12 | - | 1.85 | 0.005 | 1.44 | 1.12 | - | 1.86 | 0.005 |
|  | G5 | 2.29 | 1.60 | - | 3.29 | <0.001 | 2.32 | 1.61 | - | 3.33 | <0.001 |
| Smoking status | Non-smoker | Reference |  |  |  |  | Reference |  |  |  |  |
|  | Current/past smoker | 0.86 | 0.66 | - | 1.14 | 0.30 | 0.87 | 0.66 | - | 1.14 | 0.31 |
| Dehydration | | 1.58 | 1.21 | - | 2.06 | 0.001 | 1.56 | 1.19 | - | 2.04 | 0.001 |
| Respiratory failure | None | Reference |  |  |  |  | Reference |  |  |  |  |
|  | Moderate | 1.87 | 1.45 | - | 2.40 | <0.001 | 1.86 | 1.45 | - | 2.40 | <0.001 |
|  | Severe | 3.63 | 2.73 | - | 4.83 | <0.001 | 3.63 | 2.72 | - | 4.83 | <0.001 |
| Orientation disturbance | | 2.33 | 1.84 | - | 2.94 | <0.001 | 2.26 | 1.79 | - | 2.86 | <0.001 |
| Immunosuppression | | 1.73 | 1.32 | - | 2.27 | <0.001 | 1.73 | 1.32 | - | 2.27 | <0.001 |
| Pulmonary consolidation | | 1.49 | 1.18 | - | 1.87 | 0.001 | 1.49 | 1.18 | - | 1.87 | 0.001 |
| Hypotension | | 1.55 | 1.14 | - | 2.10 | 0.005 | 1.52 | 1.12 | - | 2.06 | 0.008 |
| Pneumonia type | Community-acquired | Reference |  |  |  |  | Reference |  |  |  |  |
|  | Nursing and healthcare-associated | 1.47 | 1.01 | - | 2.15 | 0.044 | 1.46 | 1.00 | - | 2.14 | 0.047 |
| Charlson comorbidity index | | 1.08 | 1.01 | - | 1.15 | 0.021 | 1.08 | 1.01 | - | 1.15 | 0.017 |

BMI, body mass index; CKD, chronic kidney disease.

This multivariable regression analysis was performed by using multiple imputation for missing values in body mass index and smoking status. Length of stay is summarized/calculated for those in whom in-hospital death did not occur.
