# Supplementary material for: Impact of body mass index on in-hospital mortality in older patients hospitalized for bacterial pneumonia with non-dialysis-dependent chronic kidney disease
Source: BMC Geriatr. 2022 Dec 9;22:950. doi: 10.1186/s12877-022-03659-3 (PMC9733221; doi:10.1186/s12877-022-03659-3)
Supplement: Supplementary file 7 — Additional file 7: Table 7. Odds ratios for in-hospital mortality for covariates in the multivariable regression analysis (sensitivity analysis 4). [file 12877_2022_3659_MOESM7_ESM.docx]

**Supplementary Table 7. Odds ratios for in-hospital mortality for covariates in the multivariable regression analysis (sensitivity analysis 4).**

| Variable | Category | Model with body mass index as a categorical variable | | | | | Model with body mass index as a nonlinear continuous variable | | | | |
| --- | --- | --- | --- | --- | --- | --- | --- | --- | --- | --- | --- |
|  |  | Odds ratio | 95% Confidence interval | | | *P* value | Odds ratio | 95% Confidence interval | | | *P* value |
| Age (10-year intervals) | | 1.49 | 1.25 | - | 1.76 | <0.001 | 1.49 | 1.26 | - | 1.77 | <0.001 |
| Sex | Female | Reference |  |  |  |  | Reference |  |  |  |  |
|  | Male | 0.80 | 0.60 | - | 1.07 | 0.14 | 0.78 | 0.58 | - | 1.04 | 0.095 |
| CKD stage | G3 | Reference |  |  |  |  |  |  |  |  |  |
|  | G4 | 1.23 | 0.91 | - | 1.68 | 0.18 | 1.23 | 0.91 | - | 1.68 | 0.18 |
|  | G5 | 2.08 | 1.35 | - | 3.21 | 0.001 | 2.10 | 1.36 | - | 3.24 | 0.001 |
| Smoking status | Non-smoker | Reference |  |  |  |  | Reference |  |  |  |  |
|  | Current/past smoker | 0.71 | 0.52 | - | 0.97 | 0.029 | 0.71 | 0.52 | - | 0.97 | 0.032 |
| Dehydration | | 1.57 | 1.15 | - | 2.13 | 0.004 | 1.54 | 1.13 | - | 2.10 | 0.006 |
| Respiratory failure | None | Reference |  |  |  |  | Reference |  |  |  |  |
|  | Moderate | 1.79 | 1.33 | - | 2.41 | <0.001 | 1.79 | 1.33 | - | 2.41 | <0.001 |
|  | Severe | 3.71 | 2.67 | - | 5.15 | <0.001 | 3.70 | 2.66 | - | 5.14 | <0.001 |
| Orientation disturbance | | 2.58 | 1.97 | - | 3.38 | <0.001 | 2.49 | 1.90 | - | 3.26 | <0.001 |
| Immunosuppression | | 1.54 | 1.12 | - | 2.12 | 0.007 | 1.55 | 1.12 | - | 2.13 | 0.008 |
| Pulmonary consolidation | | 1.52 | 1.16 | - | 1.98 | 0.002 | 1.52 | 1.17 | - | 1.99 | 0.002 |
| Hypotension | | 1.50 | 1.03 | - | 2.17 | 0.034 | 1.47 | 1.01 | - | 2.13 | 0.043 |
| Pneumonia type | Community-acquired | Reference |  |  |  |  | Reference |  |  |  |  |
|  | Nursing and healthcare-associated | 1.50 | 0.99 | - | 2.27 | 0.055 | 1.48 | 0.98 | - | 2.24 | 0.064 |
| Charlson comorbidity index | | 1.11 | 1.03 | - | 1.19 | 0.005 | 1.11 | 1.04 | - | 1.20 | 0.003 |

BMI, body mass index; CKD, chronic kidney disease.

This multivariable regression analysis was performed after exclusion of patients with complications of acute kidney injury on admission. Length of stay is summarized/calculated for those in whom in-hospital death did not occur.
